# Ewing’s Sarcoma in Scapular Region

**Published:** 2011-11-27

**Authors:** S H Waqar, M A Zahid

**Affiliations:** Department of General Surgery, Pakistan Institute of Medical Sciences Islamabad, Pakistan

**Keywords:** Ewing’s sarcoma, Extra-osseous, Chemotherapy

## Abstract

Ewing's sarcoma (ES) primarily affects bones and commonly presents in adolescents and young adults. This paper reports a rare case of extra osseous ES of the scapular region in a 9 years old girl. She was treated by a multidisciplinary approach including surgery, chemotherapy and radiotherapy. She was followed up for two years and remained well.

## INTRODUCTION

ES is a rare and highly malignant small round cell tumor that primarily affects the skeletal system. In primary extra osseous ESs of soft tissue underlying bone involvement is not found. James Ewing described it in 1921 as a tumor arising from undifferentiated osseous mesenchymal cells; however, recent studies suggest that Ewing’s tumor may be of neuroectodermal origin being derived from the primitive neural tissue. It accounts for 4 to10% of all types of bone cancers, with long bones and pelvis being the most common sites. It is the second most common bone tumour of childhood and adolescence, with male preponderance of 1.6:1. It is rarely seen before the age of 5 years and after the age of 30 years [1, 2, 3, 4].

The ES usually arises in the metaphysis or diaphysis of long bones of extremities. The lungs, bones and bone marrow are the most common sites of metastasis. An extensive review of the literature showed only few reported cases of the extra-osseous ES in patients under the age of 10 years. This report describes a case of extra osseous ES of the scapular region in a 9 years old girl. This case elucidates the importance of professional knowledge of the relevant aspects of ES.

## CASE REPORT

A 9 year old girl presented in the surgical outpatient department with history of a progressive swelling over right scapular region for the last three years. Swelling started as a small lump that increased in size during last six months. Swelling was not associated with fever, malaise and fatigue. There was no history of exposure to any carcinogenic agent or radiation. Past history was not significant. It was her school teacher who asked her parents to seek medical advise for the swelling, as she was facing difficulty in writing.

The general examination of the child was normal. Local examination revealed a globular non tender swelling over the right scapular region, measuring 40x36x38 cm, having firm to hard consistency. It was mobile with well defined margins and not attached to deeper structures. Overlying skin was mobile, shiny with multiple visible vessels and a small ulcer noted in the center of the swelling (Fig. 1). There was no neurovascular deficit distal to the tumour. 

**Figure:**
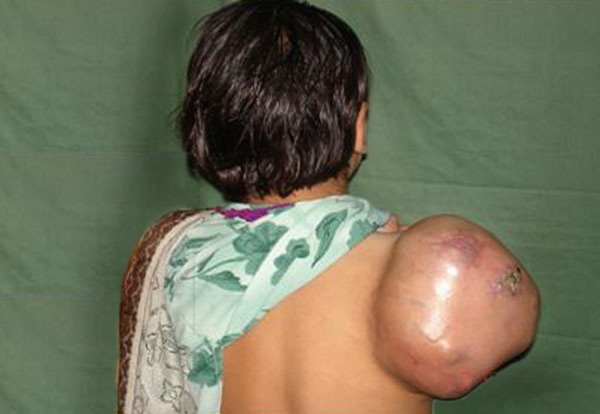
Figure 1: Mass at Scapular Region

Blood complete picture revealed mild anemia. Other blood tests including renal function test, liver function test, and serum calcium and serum alkaline phosphatase were within normal limits. X-ray and CT scan of the scapular region showed soft tissue swelling without any bony involvement (Fig. 2, 3). Fine needle aspiration suggested malignant soft tissue tumour. Incisional biopsy confirmed the diagnosis of ES. The lesion was excised and residual defect was left as such with a plan skin grafting at a later stage. The recovery was smooth and patient was discharged on fifth postoperative day. Histopathology confirmed the diagnosis of ES. All resection margins were free of tumour. Immunohistochemistry was not done. She did not report back for follow up. After three months of surgery she attended the surgical outpatient department with almost healed wound (Fig. 4). She was referred to oncologist for chemotherapy. She received VAC regimen (vincristine, adriamycin and cyclophosphamide), repeated every 3 weeks for 6 cycles and local radiotherapy was also given to the excised area. The patient responded well to treatment and has not shown any recurrence after two years of follow up. 

**Figure:**
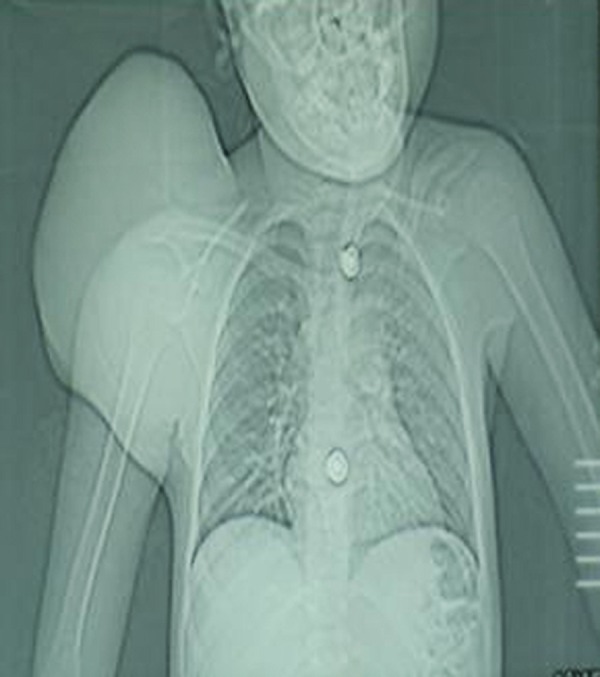
Figure 2: CT scout view showing soft tissue shadow

**Figure:**
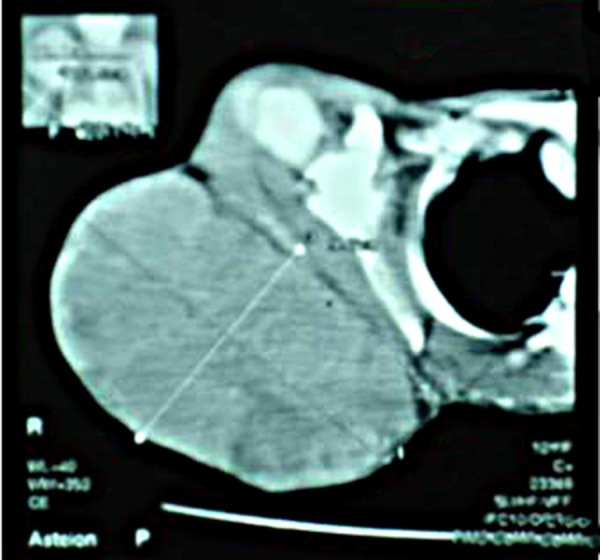
Figure 3: CT scan showing soft tissue tumour

**Figure:**
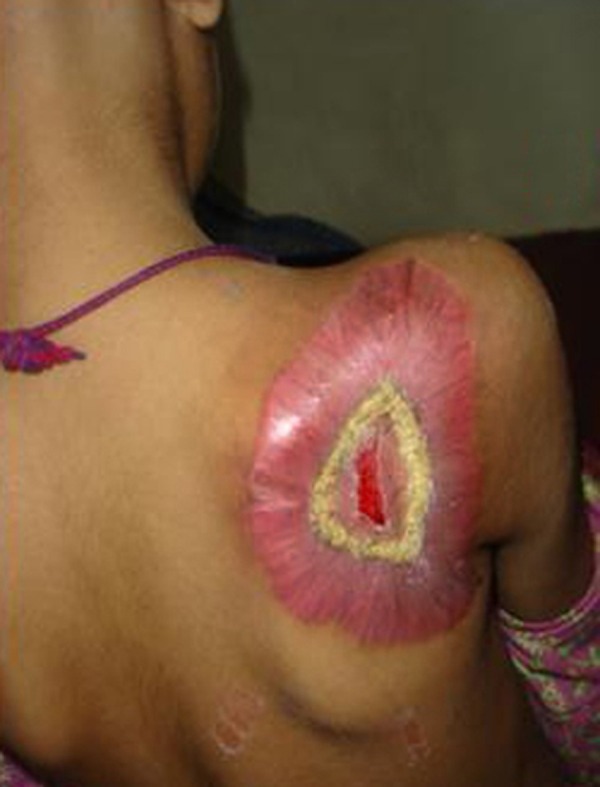
Figure 4: Three months postoperatively

## DISCUSSION

Soft tissue ES is a rapidly growing, round-cell, malignant tumour which can reach 10 cm size by the time diagnosis is made. Commonly affected extra-osseous sites are the paravertebral spaces, lower extremities, head and neck, and pelvis. One paper reported an ES involving the scapular region on literature serach. The most frequent presenting symptom is a rapidly growing mass with local pain. ES may also present with systemic signs and symptoms such as weight loss and fever [5, 6, 7]. The index case aged nine year presented with a mass without any constitutional symptoms.

Extraskeletal ES is confirmed by characteristic features on histological analysis, histochemistry, immunohistochemistry and electron microscopy. Differential diagnoses include other small, blue round cell tumours (SBRCTs) and other members of the Ewing family of tumours such as the primitive neuroectodermal tumour (PNET). Findings of X-ray and CT scan of our case showed the tumour as soft tissue swelling over scapular region. Molecular and cytogenetic analysis should be considered as the standard practice in the diagnostic evaluation of ES [5, 8].

The mainstay treatment should include multi-agent chemotherapy and aggressive surgical treatment. Tumours that are not appropriate for surgical resection or have positive margins are treated with radiation. The results of surgery alone for extra-osseous ES are poor in most of the cases, while patients receiving multimodal chemotherapy and radiotherapy have a much better prognosis. With the combination of local surgical treatment and systemic chemotherapy, long-term survival has improved from 10% to 50%-60% or greater. The prognosis for extra-osseous ES appears more favourable than that of ES in bone. More recently, Ifosfamide has emerged as a an effective chemotherapeutic agent, specially in patients resistant to other drugs. Better long term survival can be achieved in patients presenting with non metastatic disease [8, 9, 10]. Our patient responded well to the treatment and remained well after two years of follow up.

In summary, surgical resection, multi agent chemotherapy, and radio-therapy are the mainstay of treatment of ES. The treatment plan should be individualized for each patient, which should be based on age, location, stage, size of the tumor and response to therapy.

## Footnotes

**Source of Support:** Nil

**Conflict of Interest:** None declared
